# How did episiotomy rates change from 2007 to 2014? Population-based study in France

**DOI:** 10.1186/s12884-018-1747-8

**Published:** 2018-06-04

**Authors:** Karine Goueslard, Jonathan Cottenet, Adrien Roussot, Christophe Clesse, Paul Sagot, Catherine Quantin

**Affiliations:** 1 Biostatistics and Bioinformatics (DIM), University Hospital, Dijon, France; Bourgogne Franche-Comté University, Dijon, France; 2 Laboratoire interpsy (EA4432), université de Lorraine, Nancy 2, 3, place Godeffroy-de-Bouillon, 54000 Nancy, France; 3 Centre hospitalier de Jury-les-Metz, route d’Ars-Laquenexy, 57073 Jury-Les-Metz cedex 03, BP 75088 Nancy, France; 4 Gynecology Obstetrics Center, François-Mitterrand Hospital, 14, rue Paul-Gaffarel, 21000 Dijon, France; 5 Inserm, CIC 1432, Dijon, France; Dijon University Hospital, Clinical Investigation Center, clinical epidemiology/ clinical trials unit, Dijon, France; 6 Inserm, CIC 1432, Dijon, France ; Dijon University Hospital, Clinical Investigation Center, clinical epidemiology/ clinical trials unit, Dijon, France

**Keywords:** Episiotomy, Birth, Vaginal deliveries, Hospital discharge abstracts

## Abstract

**Background:**

Since the 2000s, selective episiotomy has been systematically recommended worldwide. In France, the recommended episiotomy rate in vaginal deliveries is less than 30%. The aims of this study were to describe the evolution of episiotomy rates between 2007 and 2014, especially for vaginal deliveries without instrumental assistance and to assess individual characteristics and birth environment factors associated with episiotomy.

**Methods:**

This population-based study included all hospital discharge abstracts for all deliveries in France from 2007 to 2014. The use of episiotomy in vaginal deliveries was identified by one code in the French Common Classification of Medical Procedures. The episiotomy rate per department and its evolution is described from 2007 to 2014. A mixed model was used to assess associations with episiotomy for non-operative vaginal deliveries and the risk factors related to the women’s characteristics and the birth environment.

**Results:**

There were approximately 540,000 non-operative vaginal deliveries per year, in the study period. The national episiotomy rate for vaginal deliveries overall significantly decreased from 26.7% in 2007 to 19.9% in 2014. For non-operative deliveries, this rate fell from 21.1% to 14.1%. For the latter, the use of episiotomy was significantly associated with breech vaginal delivery (aOR = 1.27 [1.23–1.30]), epidural analgesia (aOR = 1.45 [1.43–1.47]), non-reassuring fetal heart rate (aOR = 1.47 [1.47–1.49]), and giving birth for the first time (aOR = 3.85 [3.84–4.00]).

**Conclusions:**

The episiotomy rate decreased throughout France, for vaginal deliveries overall and for non-operative vaginal deliveries. This decrease is probably due to proactive changes in practices to restrict the number of episiotomies, which should be performed only if beneficial to the mother and the infant.

**Electronic supplementary material:**

The online version of this article (10.1186/s12884-018-1747-8) contains supplementary material, which is available to authorized users.

## Background

Historically, episiotomy is a surgical incision of the vaginal orifice performed to reduce severe perineal tears, per partum fetal asphyxia during the fetal expulsion stage of birth and subsequent urinary or fecal incontinence [1–3].

In the 1980s–1990s, episiotomy was routinely performed. In France, in 1998, the rate of episiotomy, usually mediolateral, was 71.3% for primiparous women and 36.3% for multiparous women [4]. This rate varied considerably from country to country. In Latin America from 1995 to 1998, the median episiotomy rate for primiparous women was 92.3% [5]. In 2000–2001, episiotomy rates were 23.8% in Canada and 32.7% in the United States while it was 100% in Taiwan in 2002. In Europe, the rates varied widely: 9.7% in Sweden in 1999–2000, 12.0% in Denmark, 13.0% in England, 44.4% in Germany in 2002–2003, and 58.0% in Italy in 1999 [6].

Since 1990, randomized controlled trials have questioned the routine use of episiotomy, which does not seem to provide more benefits than a selective practice. Indeed, restrictive episiotomy practice was associated with a higher risk of anterior perineal trauma but not with perineal infection, moderate or severe pain, long-term dyspareunia or long-term urinary incontinence, or an Apgar score less than seven at five minutes in the newborn child [3, 7–11]. Currently, selective episiotomy practice is systematically recommended, and some authors and policy statements state that episiotomy should be avoided if at all possible [10–13]. However, guidelines are relatively disparate regarding the most appropriate episiotomy rate. In 1992, Henriksen suggested an appropriate rate of 20% [14]. In 1996, the World Health Organization recommended a target rate of 10% [15]. Furthermore, the effective implementation of evidence-based healthcare practices remains a significant challenge. In 2005, as the episiotomy rate had already reached 41.3% [16], the French National College of Gynecologists and Obstetricians recommended a more restrictive practice based on « the clinical expertise of the physician », at less than 30% of vaginal deliveries [17].

To our knowledge, no studies have examined the recent trend in episiotomy rates in France as a whole even though the guidelines are about 12 years old. The aim of the present study was to describe the evolution of episiotomy rates from 2007 to 2014, especially for non-operative vaginal deliveries. We also studied the clinical and birth environment factors associated with episiotomy.

## Methods

In this population-based retrospective cohort study, we included all hospital discharge abstracts for all deliveries in France from 2007 to 2014. Diagnoses were coded according to the International Classification of Diseases (ICD-10) and procedures according to the French Common Classification of Medical Procedures (CCMP).

### Population

All hospital discharge abstracts mentioning the codes Z37 (“outcome of delivery”) of the ICD-10 were selected. In France, Z37 codes are considered the most reliable and exhaustive to select hospital deliveries. All vaginal deliveries were examined, but we especially focused on non-operative vaginal deliveries (the related codes are described in the Additional file 1).

### Variables

In the present study, the outcome of interest was episiotomy, which was defined by one code in the CCMP (JMPA006). The quality of hospital discharge abstracts regarding episiotomy has been validated thanks to a French validation study [18]. Unfortunately, to our knowledge, no validity studies have used national data to study episiotomy in non-operative vaginal deliveries.

The explanatory variables concerned the characteristics of the women and the characteristics of birth environment. At the maternal level, we considered the known risks factors for episiotomy: maternal age, parity (primiparous women were women who gave birth for the first time), gestational age (available only since 2010), multiple pregnancy, epidural analgesia, non-reassuring fetal heart rate, breech vaginal delivery, newborn weight > 4000 g (for single pregnancies, linkage with the birth abstract reliable only since 2013). We also considered severe perineal tear: third degree (injury to the anal sphincter complex) and fourth degree (injury to the perineum involving the anal, or sphincter complex and anal epithelium) [8]. Variables retained for the environment were the year of delivery, and department (French regions are divided into geographical departments).

### Statistical analysis

The characteristics are presented as means or proportions. Percentages were compared using Pearson’s Chi 2 test or Fisher’s exact test under the conditions of application. To evaluate trends in episiotomy rates between 2007 and 2014, we used the Cochran-Armitage test.

Episiotomy rates in 2007 and 2014 are presented per department (97 departments, including overseas territories).

A mixed model was used to assess associations with risk factors. As the independence of the observations could not be confirmed, hierarchical logistic regression, which took into account the hierarchical structure of data, was performed using the individual maternal variables as level 1 data, and the hospital as level 2 data.

Multilevel analyses were performed using SAS 9.4. Geographic Information System MapInfo 11.0 was used for the cartography.

This study was approved by the French Committee for Data Protection (Commission Nationale de l’Informatique et des Libertés, registration number 1576793) and was conducted in accordance with French legislation. Written consent was not needed for this study. The national hospital database was transmitted by the national agency for the management of hospitalization data (ATIH number 2015–111111–47-33).

## Results

There were about 800,000 deliveries per year (minimum 790,994 in 2007 and maximum 815,396 in 2010). The percentage of vaginal deliveries was very stable, ranging from 79.74% in 2007 to 79.59% in 2014. The proportion of non-operative vaginal deliveries slightly decreased from 68.59% in 2007 to 67.57% in 2014.

For all vaginal deliveries or for non-operative vaginal deliveries, the nationwide episiotomy rate significantly decreased (*p* < 0.01) from 26.7% (21.1% for non-operative vaginal deliveries) in 2007 to 19.9% (respectively 14.1%) in 2014. This was also the case for episiotomy rates in instrumental deliveries (61.0% in 2007 vs 52.6% in 2014, *p* < 0.01).

Then, we focused on non-operative vaginal deliveries. The characteristics of women are presented in Table 1. For women who underwent non-operative vaginal deliveries, the average maternal age was 29–30 years (+/− 5 years), slightly more than one third of women were primiparous women and about 93% of women had given birth at 37 to 41 weeks of amenorrhea (2010–2014).

**Table 1:** Characteristics of women who underwent non-operative vaginal deliveries

	2007		2008		2009		2010		2011		2012		2013		2014	
	Episiotomy	No episiotomy	Episiotomy	No episiotomy	Episiotomy	No episiotomy	Episiotomy	No episiotomy	Episiotomy	No episiotomy	Episiotomy	No episiotomy	Episiotomy	No episiotomy	Episiotomy	No episiotomy
Maternal agel (years)	29 +/− 5	30 +/− 5	29 +/− 5	30 +/− 5	29 +/− 5	30 +/−5	29 +/− 5	30 +/−5	29 +/− 5	30 +/− 5	29 +/− 5	30 +/− 5	29 +/− 5	30 +/− 5	29 +/− 5	30 +/− 5
Primiparous women ^* £^	60.6	31.6	61.3	32	62.7	32.7	63.8	32.8	63.9	32.1	64.1	31.7	64.1	31.5	64,0	31.6
Multiple pregnancies^£^	1.1	0.8	1.1	0.8	1.1	0.7	1.1	0.8	1.1	0.8	1.1	0.8	1,0	0.8	1.1	0.8
Breech vaginal delivery ^£^	2.1	1.2	2.1	1.3	2.1	1.3	2.2	1.3	2.2	1.4	2.2	1.4	2.3	1.5	2.4	1.5
Perineal tears ^£^ (third and fourth degrees)	0.4	0.2	0.5	0.3	0.5	0.3	0.5	0.3	0.6	0.3	0.6	0.3	0.7	0.4	0.8	0.4
Gestational age																
< 37 WA£	–		–		–		4.1	6.1	4	6.1	3.9	6.2	3.9	6.2	3.9	6.1
37–41 WA£	–		–		–		95.1	93.3	95.1	93.2	95.2	93,0	95.2	93.1	95.2	93.2
> 41 WA£	–		–		–		0.8	0.6	0.9	0.7	0.9	0.8	0.9	0.7	0.9	0.7

*WA* = weeks of amenorrhea, * Missing Data < 1,5%

^£^ *p* < 0.01

**Fig. 1:**
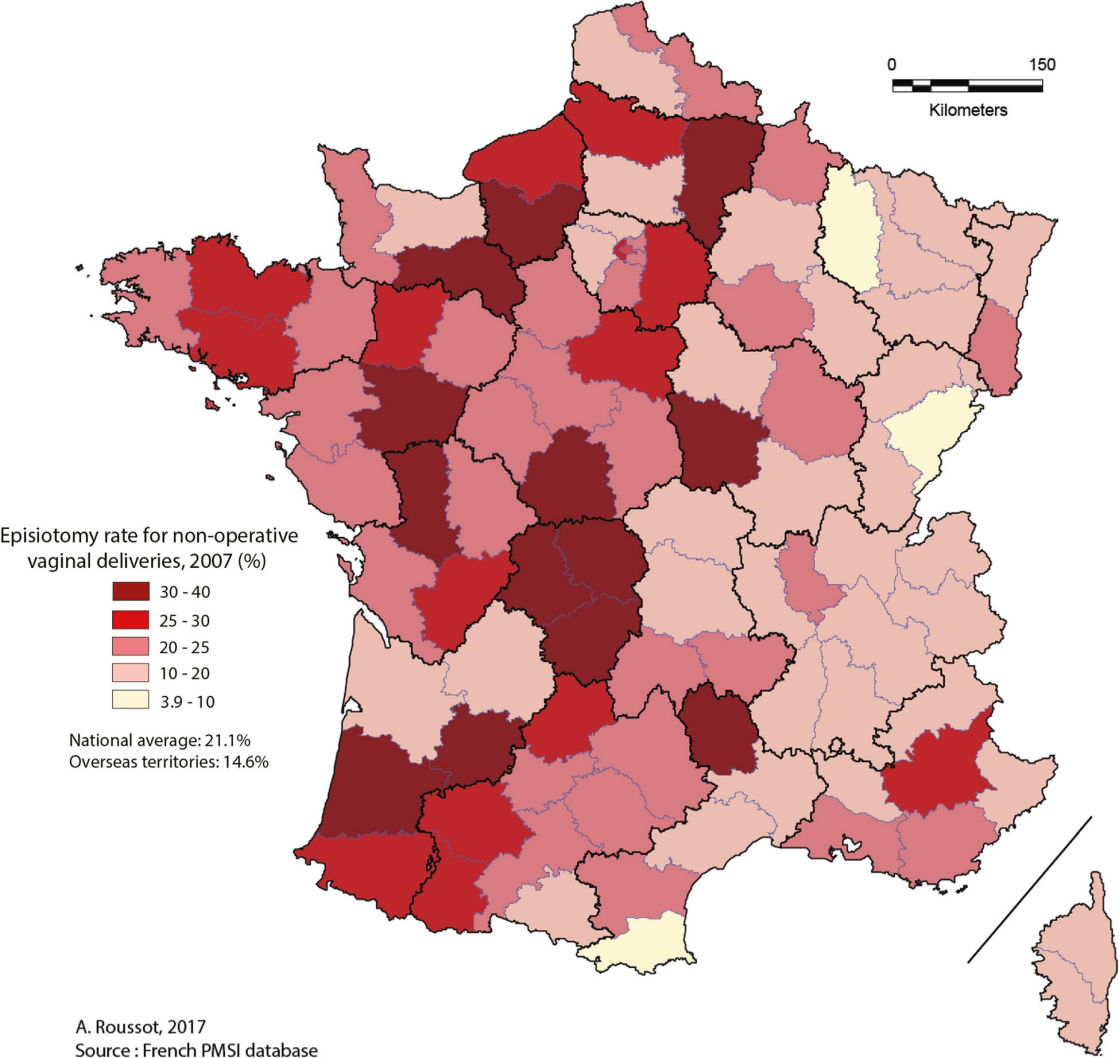
Distribution of episiotomy rates for non-operative vaginal deliveries in 2007

**Fig. 2:**
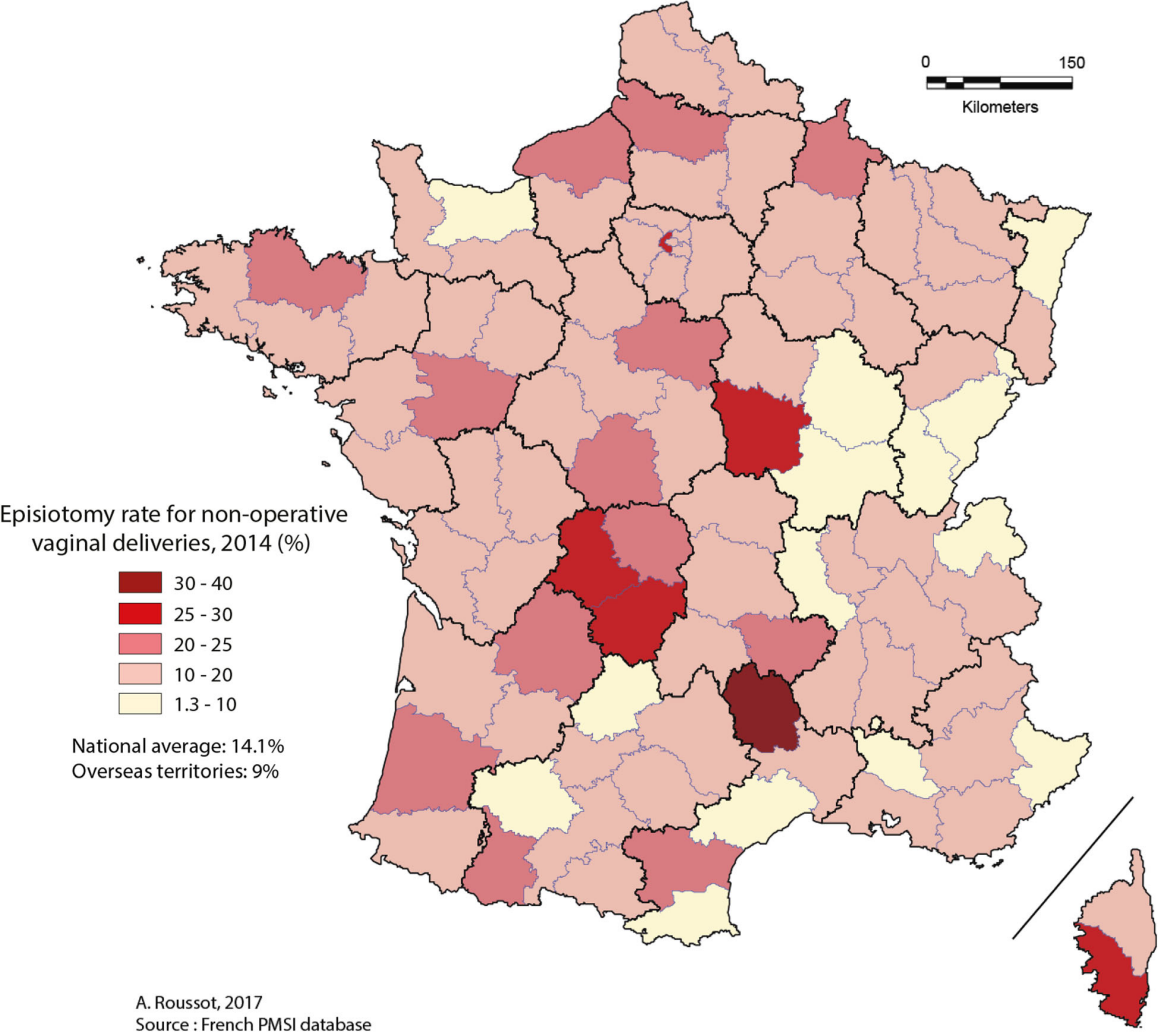
Distribution of episiotomy rates for non-operative vaginal deliveries in 2014

**Fig. 3:**
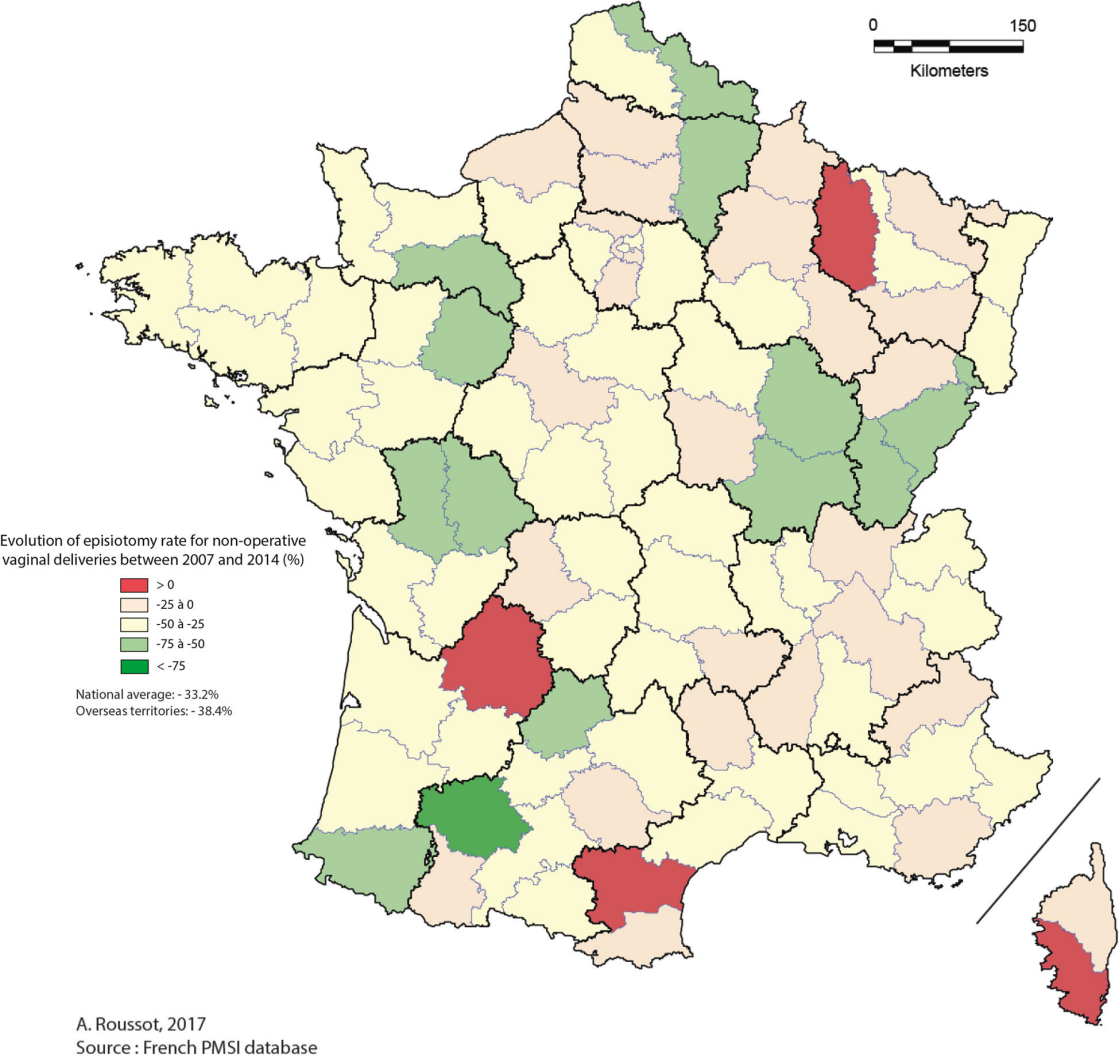
Evolution of episiotomy rates for non-operative vaginal deliveries from 2007 to 2014

For non-operative vaginal deliveries, Figs. 1 and 2 present the distribution of episiotomy rates per department in 2007 and 2014, respectively. Inter-departmental disparity was as high in 2007 (ranging from 4.0% to 39.9%) as in 2014 (ranging from 1.4% to 33.9%). The episiotomy rate decreased by 25 to 75% from 2007 to 2014 for the majority of geographic departments as shown in Fig. 3. The episiotomy rate, which was higher than 30% in 14 departments (about 15% of the 97 departments) in 2007, reached a high rate (33.9%) in only one department in 2014.

Regarding the rate of severe perineal tears (third and fourth degree) for non-operative vaginal deliveries, we observed a significant increase between 2007 and 2014, for women with episiotomy (0.4 to 0.8%, *p* < 0.01) and without episiotomy (0.2 to 0.4%, *p* < 0.01). The distribution of severe perineal tears is presented in the Appendix (Additional file 2).

The results of the multilevel logistic regression analyses are shown in Table 2 for different periods, for non-operative vaginal deliveries. From 2007 to 2014, singleton pregnancy was associated with a decrease in the use of episiotomy (adjusted Odds Ratio (aOR) = 0.74, Confidence interval 95% [0.72–0.76]), as was maternal age under 20 years (aOR = 0.89 [0.87–0.91]). Breech vaginal delivery (aOR = 1.27 [1.23–1.30]), epidural analgesia (aOR = 1.45 [1.43–1.47]), non-reassuring fetal heart rate (aOR = 1.47 [1.47–1.49]), and giving birth for the first time (aOR = 3.85 [3.84–4.00]) were significantly associated with a higher risk of episiotomy. In addition to the significant variables mentioned above, we found that giving birth before 41 weeks of amenorrhea (from 2010 to 2014) and newborn weight lower than 4000 g (from 2013 to 2014) significantly (*p* < 0.0001) decreased the risk of episiotomy.

**Table 2:** Hierarchical logistic regressions, non-operative vaginal deliveries

	Episiotomy (2007–2014)		Episiotomy (2010–2014)		Episiotomy (2013–2014)^c^	
	aOR	95% CI	aOR	95% CI	aOR	95% CI
Maternal age (ref ≥ 40 years)						
< 20	0.89	[0.87–0.91]	0.89	[0.86–0.92]	0.85	[0.81–0.90]
20–29	1.03	[1.01–1.05]	1.03	[0.99–1.04]	0.98	[0.94–1.02]
30–39	1.09	[1.07–1.12]	1.07	[1.05–1.09]	1.04	[1.01–1.08]
Single pregnancy (ref = 0)	0.74	[0.72–0.76]	0.57	[0.55–0.59]	^a^	^a^
Breech vaginal delivery (ref = 0)	1.27	[1.23–1.30]	1.45	[1.41–1.48]	1.59	[1.49–1.67]
Epidural analgesia (ref = 0)	1.45	[1.43–1.47]	1.43	[1.43–1.45]	1.47	[1.45–1.52]
Non-reassuring fetal heart rate (ref = 0)	1.47	[1.47–1.49]	1.49	[1.47–1.52]	1.49	[1.47–1.54]
Status of health establishment (ref = Private)	1.33	[1.12–1.56]	1.30	[1.09–1.54]	1.37	[1.15–1.64]
Year of delivery (ref = 2014)						
2007	1.63	[1.61–1.64]				
2008	1.58	[1.56–1.60]	^a^	^a^	^a^	^a^
2009	1.46	[1.44-1.48]	^a^	^a^	^a^	^a^
2010	1.37	[1.36-1.39]	1.37	[1.35–1.38]	^a^	^a^
2011	1.31	[1.29-1.32]	1.30	[1.29–1.32]	^a^	^a^
2012	1.21	[1.20-1.23]	1.21	[1.20–1.23]	^a^	^a^
2013	1.13	[1.12-1.15]	1.13	[1.12–1.15]	1.13	[1.11–1.14]
Parity (ref = multiparous women)	3.85	[3.84–4.00]	4.00	[4.00–4.17]	4.17	[4.17–4.35]
Gestational age (ref > 41 WA)						
< 37WA	^b^	^b^	0.42	[0.40-0.44]	0.42	[0.39–0.45]
37-41WA	^b^	^b^	0.82	[0.79-0.85]	0.82	[0.77–0.87]
Newborn weight (ref < 4000 g)	^b^	^b^	^b^	^b^	1.54	[1.49-1.56]

aOR: Adjusted Odds ratio, CI: Confidence Interval, WA: Weeks of Amenorrhea

^a^Not studied

^b^Not available in database

^c^Only single pregnancy

As regards all vaginal deliveries, the results of the analyses were similar (Additional file 3).

## Discussion

Over the last few years, the episiotomy rate significantly decreased at national and departmental level. In 2014, for non-operative vaginal deliveries, the national rate and the rates for all of the geographic departments except one were below 30%. These results suggest that the recommendations have been seriously taken into account and that proactive changes in practices to restrict the use of episiotomy have been implemented nationwide.

One of the strengths of our study was to include nearly all deliveries, thanks to national discharge abstract data, as almost all deliveries occur in hospitals in France: the difference in the total number of deliveries when compared with the national civil registry, which records all births in France, was only 0.3% [19]. The National Perinatal Survey in 2010 showed a vaginal delivery rate of 79.0% and a non-operative vaginal delivery rate of 66.9% [4]. Our results were 79.4% for all vaginal deliveries and 67.7% for non-operative vaginal deliveries.

In 2012, at the individual level, a validation study was performed to evaluate the metrological quality of hospital discharge abstracts for perinatal indicators. The validity study concerned the same data but only from three university hospitals which agreed to provide a comparison between hospital discharge abstracts and medical records. For vaginal deliveries, the positive predictive value (PPV) was 99.5% [98.5–100] and the sensitivity (Se) was 100%. For episiotomy, irrespective of the vaginal mode of delivery, the PPV was 88.9% [79.7–98.1] and the Se was 90.9% [82.4–99.4]. For perineal tears in vaginal deliveries, the PPV was 94.3% [89.9–98.7] and the Se was 88.6% [82.8–94.4] [18]. In France, no data from validation studies are available for severe perineal tears in cases of non-operative vaginal deliveries. However, in 2010, the rate of severe perineal tears for vaginal deliveries was 0.6% in our study, while the rate was estimated at 0.8% ([0.6–0.9]) in the National Perinatal Survey [4].

Moreover, our population-based study allowed us to examine some groups like breech vaginal deliveries or multiple deliveries with large sub-populations. These national data also allowed us to take into account the effects of the health facility or the variability in hospital medical practices as we included a specific level for hospitals in our multilevel model (level 2 of the model).

The main limitation of this study was related to differences in coding practices. In the validation study in 2012, we examined divergent cases for the discussion. During interviews, we were able to discern that some physicians could seldom report the code for episiotomy in cases of delivery with instrumental assistance, considering that episiotomy is a classical part of this delivery procedure. For this reason, we decided to focus on non-operative vaginal deliveries. All tables and figures were restricted to these cases. The results for all vaginal deliveries are only given for information (Additional file 3), as we know that they may be underestimated. However, when an episiotomy is coded, this episiotomy is generally performed.

Our results are consistent with previous studies regarding factors associated with the use of episiotomy. We retrieved the usual risk factors: primiparous women, multiple pregnancies, breech vaginal deliveries, epidural analgesia, non-reassuring fetal heart rate, newborn weight > 4000 g [20–23]. In France, episiotomy is not systematically used in breech vaginal deliveries. Indeed, the episiotomy rate decreased from 57% in 1994 to 28.4% in 2009–2010 [24]. Although the restrictive practice of episiotomy has been established by evidence-based medicine, the indications to perform episiotomy are still a matter of debate.

In France, even though, to our knowledge, no national rates have been published for all hospitals, some studies have been performed, and their results seem to agree with ours. A first study in 2007, which concerned vaginal deliveries in university hospitals, estimated a national episiotomy rate of 32.4%. It is not surprising that our estimation for vaginal deliveries in 2007 (26.7%) was lower as we considered all hospital types [25]. In 2009, another study in one hospital estimated the episiotomy rate in vaginal deliveries at 7.6%. This figure is slightly below our estimation (11.3%, all over the corresponding department) but the 2009 study included only one hospital and only single pregnancies and cephalic presentations [26]. Another study based on Burgundy Perinatal Network data showed similar results to ours in the four departments included in this region [27]. A study conducted in the south of France reported a decrease in the rate of episiotomy (from 35.8% in 2003–2005 to 16.7% in 2012–2014) [20]. All these studies highlight a high disparity in episiotomy rates not only between departments but also between hospitals. In our study, a decrease in episiotomy rates was shown for the vast majority of French departments from 2007 to 2014. Over the same period, severe perineal tears (third and fourth degrees) significantly increased in women who had non-operative vaginal deliveries. These results were consistent with those of the Euro-peristat project, which described an increase in the rate of severe perineal tears for all vaginal deliveries between 2004 and 2010 in all European countries, except Germany and Norway [28]. This issue is still the subject of a controversial debate [7, 9, 20, 29–32]. Randomized trials showed no increase in severe perineal tears related to the restrictive use of episiotomy [3]. On the contrary our results showed that the restrictive practice of episiotomy was associated with a greater risk of severe perineal tears. However, the rate in France is lower than the mean rate found in EURO-PERISTAT, which suggests that the rate is under-estimated in France. Given that the rate found in our study is in keeping with that found in the national perinatal survey, it is likely that this under-estimation does not stem from coding problems in hospital data. Another explanation could be non-diagnosis [33], which nonetheless seems to be diminishing over time. In fact, we observed an increase in severe perineal tears in France which may be related to improvements in vigilance and the training of professionals in the diagnosis and suturing of these severe perineal tears which are responsible for urinary and fecal incontinence [29].

We can notice that, for non-operative vaginal deliveries, the rate decreased markedly in all departments (decrease of 25 to 75%), even when initial rates were about 30%. In 2014, 14 of the 97 departments presented an episiotomy rate below 10% for non-operative vaginal deliveries. The rate of severe perineal tears was unavailable for two of these departments, between 0.15 and 0.58% for ten departments and above 1% for two departments.

It seems difficult to define what could be the right episiotomy rate in France. The WHO recommended a target of 10% for episiotomy. This recommendation cannot be generalized as it was based on a case-controlled study that included only non-induced labor for a single pregnancy at over 37 weeks of amenorrhea. Moreover, it took into account the high infection rate in developing countries, which is not the case in France. The restrictive practice of episiotomy must provide an episiotomy rate that is optimal for children’s and mothers’ health and ensure low rates of severe perineal tears, which are very harmful for women. Our results suggest that a rate below 15% for non-operative vaginal deliveries was obtained in 57% of French departments with a rate of severe perineal tears not more than 1%. As a consequence, one could hypothesize than a rate of 15% could be reached by most departments in a reasonable time. Further research is of course needed to confirm this hypothesis.

This target rate can be considered achievable for all French departments, though a national program is necessary. A passive approach after the publication of guidelines is not enough and the implementation of evidence-based practices remains a real challenge. Previous publications have shown that the impact of guidelines is greater if they are worked on with the teams concerned, particularly in obstetrics [34]. At the national level, a community of practices could promote the dissemination of experience, and thus decrease the episiotomy rate without increasing severe perineal tears.

Our study suggests that the action plan should now be looking at the individual level. Some authors have described how a private and confidential feedback from physicians about their own practices can induce a decrease in the use of episiotomy [35, 36]. Ambassadors with communication and training skills may effectively facilitate changes in their teams [37]. In the same way, audits, risk management approaches, continuous care quality improvement programs and the understanding of professionals’ behavior with regard to perineum protection should lead to the standardization of good practices for selective episiotomy.

The continuous training of physicians and midwives is an important lever to improve quality of care. Perinatal networks, which aim to inform, train and motivate practitioners, gain their support in implementing a restrictive practice policy for episiotomy. Perinatal networks have a role to play in standardizing the restrictive use of episiotomy in the areas they cover.

## Conclusions

Our study described the evolution of episiotomy rates in France following the recommendations of the National College of Gynecologists and Obstetricians. We showed that the use of episiotomy in non-operative vaginal deliveries fell significantly from 21.1% in 2007 to 14.1% in 2014 at the national and departmental level. However, at the departmental level, the episiotomy rates still ranged from 1.4% to 33.9%. To reduce the still present disparities without impairing the health of women and their children, it is necessary to act at the individual level and to encourage every professional to think about his/her use of episiotomy in the light of clear, relevant indicators.

## Additional files

Additional file 1: ICD-10 codes and CCMP codes. Description of codes used for identification of delivery modes and risk factors. (DOCX 14 kb)

Additional file 2: Figure: Distribution of severe perineal tears rates for non-operative vaginal deliveries in 2014. The figure presents the rate of severe perineal tears per department, in 2014, in France. (DOCX 246 kb)

Additional file 3: Table: Hierarchical logistic regressions, all vaginal deliveries. The table presents the association between the episiotomy and risk factors, for all vaginal deliveries. (DOCX 18 kb)

## Abbreviations

aOR: Adjusted Odds Ratio
CCMP: Common Classification of Medical Procedures
CI: Confidence Interval
ICD-10: International Classification of Diseases-version10
PPV: Positive Predictive Value
Se: Sensitivity
WA: Weeks of Amenorrhea
