# Supplementary material for: How did episiotomy rates change from 2007 to 2014? Population-based study in France
Source: BMC Pregnancy Childbirth. 2018 Jun 4;18:208. doi: 10.1186/s12884-018-1747-8 (PMC5987447; doi:10.1186/s12884-018-1747-8)
Supplement: Supplementary file 1 — ICD-10 codes and CCMP codes. Description of codes used for identification of delivery modes and risk factors. (DOCX 14 kb) [file 12884_2018_1747_MOESM1_ESM.docx]

Appendix

*ICD-10 codes and CCMP codes*

Vaginal deliveries - ICD-10 codes: O80, O81, O83, O84 and CCMP codes: JQGD010, JQGD012

Non-operative vaginal deliveries - ICD-10 code: O80

Multiple pregnancies - ICD-10 code: O84 and CCMP codes: JQGD002, JQGD007

Epidural analgesia-CCMP code: AFLB010

Non-reassuring fetal heart rate - ICD-10 code: O68.0

Breech vaginal delivery-CCMP codes: JQGD001, JQGD003, JQGD004, JQGD005, JQGD008, JQGD013

Perineal tear third degree - ICD-10 code: O70.2

Perineal tear fourth degree - ICD-10 code: O70.3
