# Supplementary figures and images for: How did episiotomy rates change from 2007 to 2014? Population-based study in France
Source: BMC Pregnancy Childbirth. 2018 Jun 4;18:208. doi: 10.1186/s12884-018-1747-8 (PMC5987447; doi:10.1186/s12884-018-1747-8)

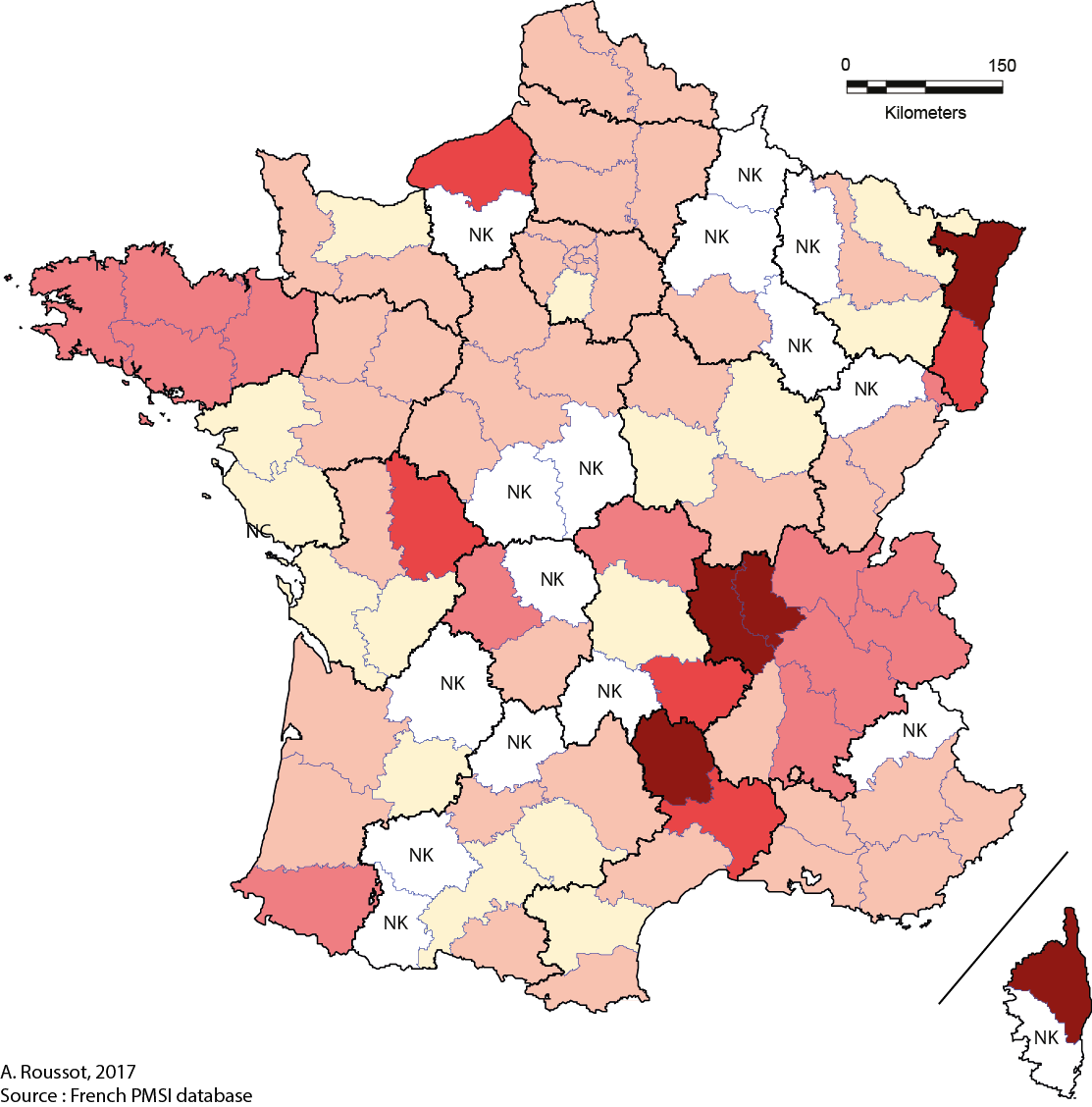


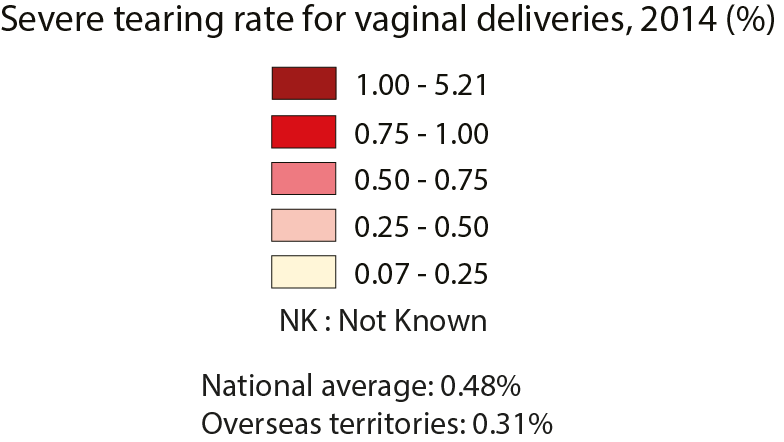

Supplement: Supplementary file 2 — Figure: Distribution of severe perineal tears rates for non-operative vaginal deliveries in 2014. The figure presents the rate of severe perineal tears per department, in 2014, in France. (DOCX 246 kb) [file 12884_2018_1747_MOESM2_ESM.docx]
