# Supplementary material for: How did episiotomy rates change from 2007 to 2014? Population-based study in France
Source: BMC Pregnancy Childbirth. 2018 Jun 4;18:208. doi: 10.1186/s12884-018-1747-8 (PMC5987447; doi:10.1186/s12884-018-1747-8)
Supplement: Supplementary file 3 — Table: Hierarchical logistic regressions, all vaginal deliveries. The table presents the association between the episiotomy and risk factors, for all vaginal deliveries. (DOCX 18 kb) [file 12884_2018_1747_MOESM3_ESM.docx]

| Hierarchical logistic regressions, all vaginal deliveries | | | | | | |
| --- | --- | --- | --- | --- | --- | --- |
|  |  |  |  |  |  |  |
|  | Episiotomy  (2007-2014) | | Episiotomy  (2010-2014) | | Episiotomy^¥^  (2013-2014) | |
|  | aOR | 95% CI | aOR | 95% CI | aOR | 95% CI |
| Maternal age (ref≥40 years) | |  |  |  |  |  |
| <20 | 0.75 | [0.73-0.76] | 0.74 | [0.72-0.76] | 0.85 | [0.81-0.90] |
| 20-29 | 0.95 | [0.93-0.96] | 0.93 | [0.91-0.94] | 0.98 | [0.94-1.02] |
| 30-39 | 1.04 | [1.02-1.06] | 1.01 | [0.99-1.03] | 1.04 | [1.00-1.08] |
| Single pregnancy (ref=0) | 0.74 | [0.72-0.75] | 0.58 | [0.56-0.60] | * | * |
| Epidural analgesia (ref=0) | 1.61 | [1.59-1.61] | 1.61 | [1.61-1.64] | 1.48 | [1.46-1.51] |
| Non-reassuring fetal heart rate (ref=0) | 1.72 | [1.72-1.75] | 1.69 | [1.69-1.72] | 1.50 | [1.46-1.54] |
| Year of delivery (ref=2014) | |  |  |  |  |  |
| 2007 | 1.63 | [1.61-1.64] |  |  |  |  |
| 2008 | 1.49 | [1.47-1.50] | * | * | * | * |
| 2009 | 1.38 | [1.36-1.39] | * | * | * | * |
| 2010 | 1.31 | [1.30-1.32] | 1.31 | [1.30-1.32] | * | * |
| 2011 | 1.26 | [1.25-1.27] | 1.26 | [1.25-1.28] | * | * |
| 2012 | 1.18 | [1.17-1.19] | 1.18 | [1.17-1.19] | * | * |
| 2013 | 1.12 | [1.10-1.13] | 1.12 | [1.11-1.13] | 1.13 | [1.11-1.14] |
| Parity (ref=multiparous women) | 4.55 | [4.55-4.55] | 4.76 | [4.76-4.76] | 4.22 | [4.17-4.27] |
| Gestational age (ref>41 WA) | |  |  |  |  |  |
| < 37WA | ** | ** | 0.38 | [0.37-0.39] | 0.42 | [0.39-0.45] |
| 37-41WA | ** | ** | 0.75 | [0.73-0.78] | 0.82 | [0.77-0.87] |
| Newborn weight (ref<4,000g) | ** | ** | ** | ** | 1.53 | [1.49-1.57] |
| aOR: Adjusted Odds ratio, CI: Confidence Interval, WA: Weeks of Amenorrhea | | | | | |  |
| * Not studied |  |  |  |  |  |  |
| ** Not available in database  ^¥^ Only single pregnancy | |  |  |  |  |  |
